# R software: unfriendly but probably the best

**DOI:** 10.3325/cmj.2020.61.66

**Published:** 2020-02

**Authors:** Branimir K. Hackenberger

**Affiliations:** Department of Biology, Josip Juraj Strossmayer University, Osijek, Croatia *hackenberger@biologija.unios.hr*

Each of us has a friend with a difficult personality. However, we would not waste our time and masochistically put up with their personality if it did not benefit us in some way. And whenever we organize a get-together we always invite this friend, even though we know in advance that it would not go smoothly. It is a similar situation with R software.

I am often asked how I can be so in love with this unfriendly software. I am often asked why R. My most common answer is: “Why not?!” I am aware of the beginners' concerns because I used to be one myself. My first encounter with R was in 2000, when I found it on a CD that came with some computer magazine. I installed it, tried it out a bit, and deleted it after a few days, pretty sure that it was forever. As an experienced user of commercial software with an attractive graphical user interface, I never thought of memorizing commands and typing them in the line editor of the R interpreter. That seemed like going on foot despite having a good car. But very soon after my first encounter with R, I began to perform more demanding statistical analyses, which required tools far more complex than ANOVA or MANOVA. At one point, I reached the end of commercial software capabilities. To solve my problem, it was necessary to create new algorithms for data preparation and analysis. In addition, I started noticing more and more often that two or three commercial software packages gave different results. As commercial programs are usually closed source, I was not able to see how their procedures differ. Then I remembered R, and after about a dozen installations and uninstallations, I started to very efficiently solve all my problems. Namely, R is open-source software. Apart from the fact that a large part of the code is visible, there is a possibility to directly contact the author of individual packages. Furthermore, at that time, around 2001, new mailing lists for R users existed and were emerging. If I had any questions not only regarding the use of R but also the statistical methods themselves, I would get an answer within hours. After a year, I used R for my entire data processing needs. The advent of excellent IDEs, first RKWard and later RStudio, made it much easier to work with R and solidified our ongoing relationship.

The biggest problem for R newbies is the knowledge and understanding of statistics. Unlike the use of commercial software, where the lists of suggested methods appear in windows or drop-down menus, the use of R requires *a priori* knowledge of the method that should be used and the way how to use it. While this may seem aggravating and unfriendly, it reduces the possibility of using statistical methods incorrectly. If one understands what one is doing, the chance of making a mistake is reduced. Here, we come to the general problem of applying statistical methods. This problem is perhaps best described by Richard McElreath in his book Statistical Rethinking (1). This book clearly describes the common practice of treating statistics as a necessary evil and likens the choice of a statistical method to the choice of dishes in a recipe book.

Learning to process data using R is already in the curriculum of many universities. Perhaps this is the case because teaching statistics with R provides complete data processing training and presents statistics as a purposeful and powerful tool. And statistics is exactly that (or only that), a powerful tool that allows us to get the most out of data. An excellent account of this attitude toward mathematics in general is outlined in the scientific essay Mathematics Is Biology's Next Microscope, Only Better; Biology Is Mathematics' Next Physics, Only Better by Joel E. Cohen (2). Mathematics and statistics, used properly, make it possible to identify relationships in a given data set that would otherwise remain invisible.

R is rewarding and demanding at the same time. It provides reliable calculations and almost immeasurable data processing capabilities, but requires at least basic knowledge of the statistical methods used.

How was R created and how did it become so popular? R was created by statistician Ross Ihaka and statistician and bioinformaticist Robert Gentleman from the University of Auckland in 1992 on the basis of the programming language S. The first official stable version (1.0) was released in 2000. Today, R is developed by the R Development Core Team. A very good overview of the development of R, as well as a description the subcultural phenomenon related to it, was made by Nick Thieme in 2018 (3).

The basic idea of John Chambers, the creator of the S programming language, was to create a piece of software that would focus on interactive data processing, ie, statistics, and at the same time have the features of a programming language, ie, enable writing and executing of programs. R inherited this concept and brought it to a higher level. R, especially RStudio, enables interactive data analysis, as well as the writing of programs and scripts with a whole range of other possibilities for creating various types of documentation. Everything from the automated creation of pdf or Word documents to the creation of interactive web pages can be done in a single program. Although R is just an interpreter, its flexibility and power is almost fantastic and can be used very successfully in applications written in C/C++ or Python. Numerous packages developed in the last few years allow the flow of data between R and other software, the use of modules written in other languages (C/C++, Python), and the use of R functions in applications written in other computer languages.

Global dissemination of R was facilitated by the Comprehensive R Archive Network (CRAN), a network of FTP and web servers that stores identical, up-to-date, code versions, and documentation about R. There are currently 94 official and active CRAN servers that contain all the software and documentation.

The power of R lies in the existence of numerous packages with functions, algorithms, and procedures for various types of data processing. Currently there are 15 359 peer-reviewed and tested packages on CRAN servers. There are several hundred more packages on numerous servers at universities and institutes. These packages have not been checked and tested but are being used locally (or can be downloaded for free from the institutions' servers). Further advantages of the program are the flexibility of the packages in terms of the language in which their functions are written (C / C ++, Fortran, Java, Python) and the redundancy of statistical methods (multiple packages contain the same methods that can be realized by different algorithms).

The first R users were often teased for using a software package “that had no future.” On internet forums, R was described as a free tool that is so unfriendly that it will disappear very quickly. However, R was getting better, and the number of users was growing almost exponentially. And, more importantly, R stayed free. How can something free be so good? This is a question that is often heard but more often remains unspoken.

The answer may lie in the fact that R is not just a piece of software. It is also a social and subcultural phenomenon. Namely, packages, which are all functions used for statistical analysis, are made by the users themselves. R development is propelled by incredible enthusiasm and altruism of hundreds of scientists who share their intellectual achievements on CRAN. The speed and amount of help that is shared daily among users through the official and unofficial user forums and networks is amazing.

Although R is completely free, activities related to this software package are extremely frequent. The members of the R Development Core Team have set up a non-profit organization, called The R Foundation, whose main mission is a continued development of R, the exploration of new methodology, teaching and training of statistical computing, and the organization of meetings and conferences on statistical computing. The first International R User Conference, also known by its short name useR!, was held in 2004 in Vienna, Austria. Since 2006, this conference has been held regularly and has been organized on three continents (Europe, North America, and Australia). In addition to this conference, there are thematic conferences such as R/Medicine and R/Pharma on the use of R in medicine and pharmacy. In 2009, The R Foundation began publishing *The R Journal*, an open access, refereed journal of the R project for statistical computing.

The beginning of the 21st century was marked by a tremendous advancement of genomics and molecular biology and the development of high-throughput technologies. The ability of these new technologies to obtain a large number of data warranted the development of new tools in computational biology and bioinformatics. This led to the emergence of the Bioconductor project. The goal of the Bioconductor was to develop a platform for storing and sharing open-source algorithms for genome-scale data analysis in biology and medicine. The Bioconductor repository currently contains over a thousand of software packages written in R for analyzing data sets of various sizes, from cDNA microarrays to copy-number variation and epigenomics. Like R, Bioconductor is completely free. In addition to other literature, an excellent overview of some capabilities within the Bioconductor has been given by Robert Gentleman (4) and Sorin Drăghicia (5).

**Figure 1:**
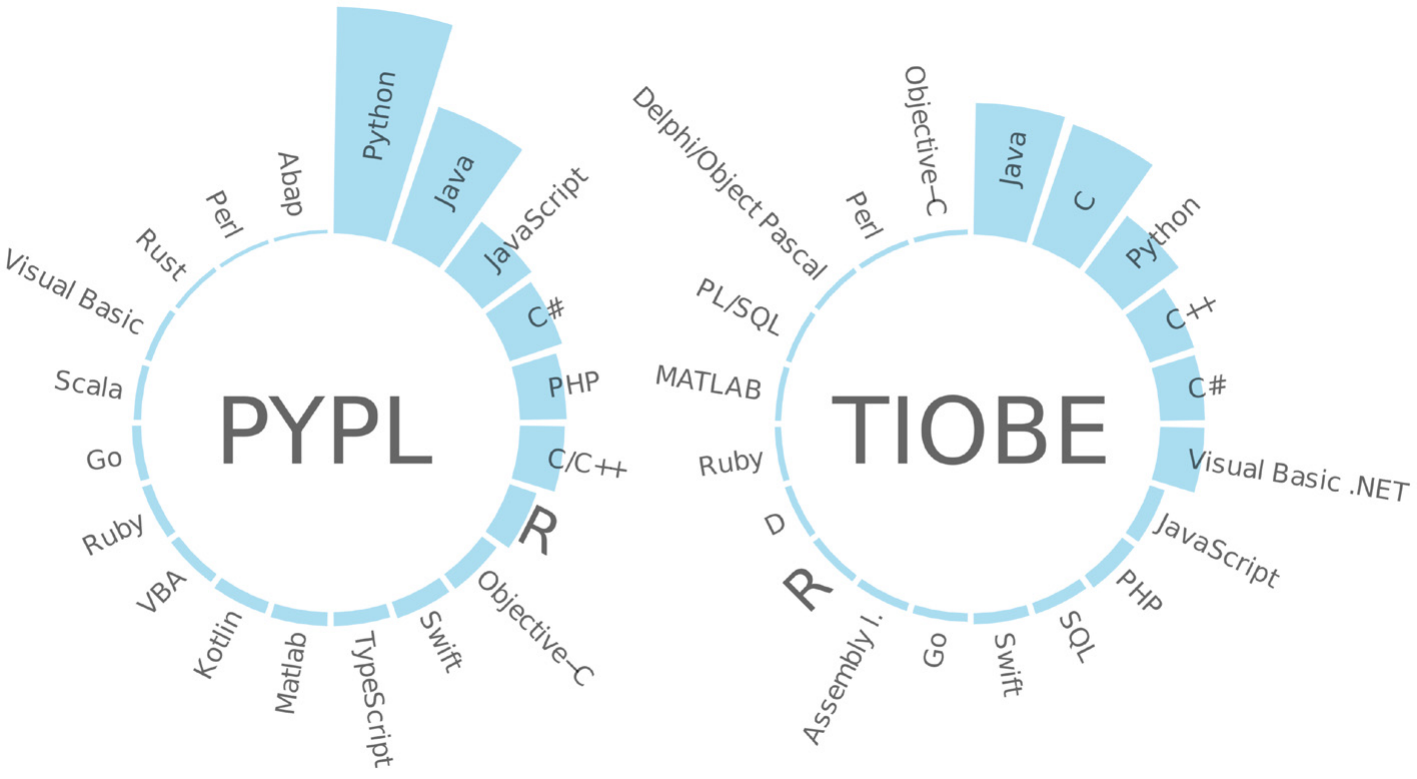
Ranking of R popularity based on TIOBE and PYPL indexes.

According to the TIOBE Popularity Index for February 2020, R ranked 13th (out of 265 programming languages) (6) and according to the PYPL (PopularitY of Programming Language) index, it ranked seventh (7) (Figure 1). The popularity of R is further increased by the fact that R is primarily and specifically a data processing, ie, statistical computer language, while other software languages are mostly languages of general and universal application.

One of the advantages a large number of users working together to develop new packages and implement the latest computer technologies is that R is able to follow the latest trends faster than competitive software. The latest developments are being incorporated into new packages very quickly and the core code is updated on average twice a year. At the moment, the R programming environment contains the most extensive range of tools for parallel computing, machine and deep learning, and for working with Big Data, including Torch and TensorFlow, greatly facilitating and accelerating the construction and implementation of neural networks. Many of the negative reviews about individual R packages soon become outdated because the package authors quickly eliminate the shortcomings.

Although the learning curve of this programming language and data processing system is steep at the beginning, the benefit gained by using R far exceeds the effort invested. Especially if learning of this computer language is combined with repeating or learning statistics. Although unfriendly, R is perhaps the today's best tool for statistical data processing, ranging from frequentist statistics, Bayesian statistics, meta-analysis, machine and deep learning to parallel computing and Big Data processing. For free!
